# Translating genotype data of 44,000 biobank participants into clinical pharmacogenetic recommendations: challenges and solutions

**DOI:** 10.1038/s41436-018-0337-5

**Published:** 2018-10-16

**Authors:** Sulev Reisberg, Kristi Krebs, Maarja Lepamets, Mart Kals, Reedik Mägi, Kristjan Metsalu, Volker M. Lauschke, Jaak Vilo, Lili Milani

**Affiliations:** 1 Institute of Computer Science, University of Tartu, Tartu, Estonia; 2 STACC, Tartu, Estonia; 3 Quretec, Tartu, Estonia; 4 Estonian Genome Center, Institute of Genomics, University of Tartu, Tartu, Estonia; 5 Institute of Molecular and Cell Biology, University of Tartu, Tartu, Estonia; 6 Department of Physiology and Pharmacology, Section of Pharmacogenetics, Karolinska Institutet, Stockholm, Sweden; 7 Science for Life Laboratory, Department of Medical Sciences, Uppsala University, Uppsala, Sweden

**Keywords:** pharmacogenetics, pharmacogenomics, biobank participants, preemptive pharmacogenetic testing, genotyping array

## Abstract

**Purpose:**

Biomedical databases combining electronic medical records and phenotypic and genomic data constitute a powerful resource for the personalization of treatment. To leverage the wealth of information provided, algorithms are required that systematically translate the contained information into treatment recommendations based on existing genotype–phenotype associations.

**Methods:**

We developed and tested algorithms for translation of preexisting genotype data of over 44,000 participants of the Estonian biobank into pharmacogenetic recommendations. We compared the results obtained by genome sequencing, exome sequencing, and genotyping using microarrays, and evaluated the impact of pharmacogenetic reporting based on drug prescription statistics in the Nordic countries and Estonia.

**Results:**

Our most striking result was that the performance of genotyping arrays is similar to that of genome sequencing, whereas exome sequencing is not suitable for pharmacogenetic predictions. Interestingly, 99.8% of all assessed individuals had a genotype associated with increased risks to at least one medication, and thereby the implementation of pharmacogenetic recommendations based on genotyping affects at least 50 daily drug doses per 1000 inhabitants.

**Conclusion:**

We find that microarrays are a cost-effective solution for creating preemptive pharmacogenetic reports, and with slight modifications, existing databases can be applied for automated pharmacogenetic decision support for clinicians.

## INTRODUCTION

Genetic variation causing interindividual differences in drug response poses major problems for pharmacological therapy and drug development. In recent decades a plethora of associations between genetic variants and treatment efficacy or adverse drug reactions have been identified.^1^ However, the implementation of clinical pharmacogenomics is lagging far behind these discoveries.^2^ Fast, accurate, and cost-effective genotyping of genes involved in drug response is a crucial first step for the implementation of pharmacogenomics in clinical care. Ideally, the genotype data should already exist in an individual’s health record at the time when personalized treatment is necessary. The currently most widely used genotyping method is the array-based interrogation of (candidate) variants. However, due to recent progress in sequencing technologies, next-generation sequencing (NGS)-based methods, such as exome sequencing (ES) and genome sequencing (GS), are becoming more prevalent. The advantage of the latter is that sequencing-based methods detect rare variants, which have been estimated to account for 30–40% of the functional variability in pharmacogenes.^3^ Currently, multiple trials that evaluate the patient benefits of preemptive pharmacogenetic genotyping using the different methodologies are being conducted.^4–6^

For the translation of genetic testing results into treatment recommendations concerted efforts have led to the publication of genotype-based guidelines, for which strong evidence links genetic polymorphisms to variability in efficacy or risk for adverse reactions.^7^ To account for the effect of allelic variation and haplotypes of genes relevant in drug response, the “star” (*) nomenclature system is most widely used.^8^ For most genes covered by guidelines from the Clinical Pharmacogenomics Implementation Consortium (CPIC), comprehensive information tables have been prepared on how to define alleles on the basis of genetic variation, which facilitates the association of diplotypes with predicted phenotypes and thus their functional interpretation.^8,9^ A collaborative effort is underway to develop a software tool (PharmCAT) for automated conversion of genotype information into CPIC guideline recommendations.^10^

Here, we provide an overview of the challenges and solutions for the translation of genotype and sequence data of 11 genes into pharmacogenetic diplotypes and recommendations for drug prescription. We leveraged genomic information of 44,448 Estonian Biobank participants genotyped by high density microarrays, ES or GS and derived pharmacogenetic recommendations based on preexisting CPIC guidelines for 32 commonly prescribed medications. We find drastic differences in the predicted outcomes across genotyping platforms and demonstrate that GS currently does not provide substantial additional actionable information regarding common pharmacogenetic alleles compared with the latest genotyping arrays. Importantly, these recommendations can be returned to biobank participants, or incorporated into their health records for the personalization of future treatment decisions.

## MATERIALS AND METHODS

### Overview of genetic data

**Fig. 1:**
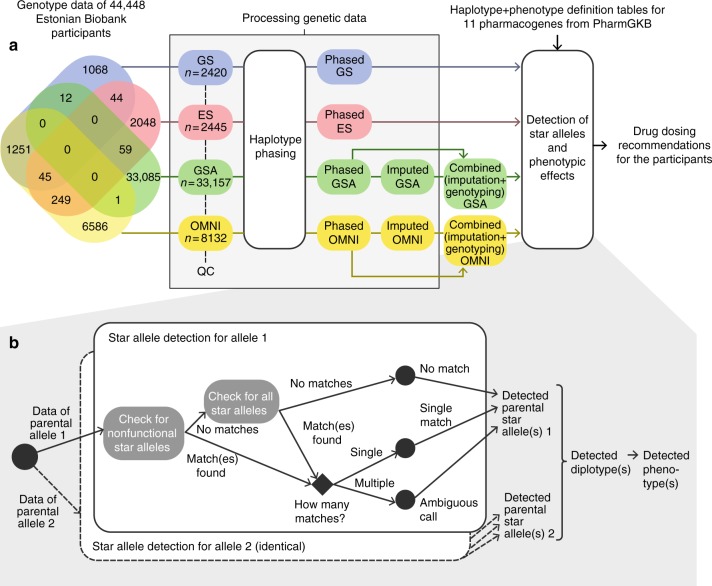
**Pipeline for extracting pharmacogenetically relevant alleles from existing genotyping data.** Panel (**a**) depicts the different data sets, their overlap (Venn diagram), and how the data were processed. Panel (**b**) zooms into the detection of star alleles according to specific definition tables. *ES* exome sequencing, *GS* genome sequencing, *GSA* Global Screening Array, *OMNI* HumanOmniExpress.

The Estonian Biobank is a research-oriented biobank containing longitudinal data and biological samples, including DNA, for 5% of the adult population of Estonia. Participants of the biobank have signed a broad informed consent that allows the Estonian Genome Center to continuously update their records through periodical linking to central electronic health record databases and local hospital information systems.^11^ Of the biobank participants, 8132 have been genotyped using the HumanOmniExpress beadchip (OMNI) and 33,157 using the Global Screening Array (GSA) from Illumina. Furthermore, ES and GS data is available for 2445 and 2420 participants, respectively (Fig. 1a). Only 1661 of the subjects (3.7%) have been genotyped on more than one platform.

For genome sequencing, DNA samples were prepared using the TruSeq PCR-free kit, and sequenced on the Illumina HiSeq X using 150 bp paired-end reads at a mean coverage of 30×. ES samples were prepared using the Agilent SureSelect Human All Exon V5 + UTRs target capture Kit according to the manufacturer’s recommendations, and sequenced on the HiSeq2500 at a mean target coverage of 67×. Details regarding the tools and parameters used for the bioinformatic analysis, including read alignment, variant calling, genotype imputation and phasing, are provided in Note S1.

The genotype data obtained on both arrays were separately phased using Eagle2 (v. 2.3) (ref. ^12^) and imputed using the BEAGLE (v. 4.1) (ref. ^13^) software implementing a joint Estonian and Finnish reference panel described in Note S1. Imputed genotypes with probabilities lower than 90% were filtered out. To call pharmacogenetic star alleles based on the microarray data we used genotyped variants together with imputed variants. In cases where the variant was both directly genotyped and imputed, the original genotype call was preferred. As a result of processing the genetic data, genetic information of all samples was converted into a joint variant call file (VCF), where variant positions were aligned against the GRCh37/hg19 human genome reference.

The genotype data is available upon request from the Estonian Biobank (https://www.geenivaramu.ee/en/biobank.ee/data-access).

### Pruning of allele definition tables

To detect star alleles, we initially set out to use entire gene-specific allele definition tables prepared by the curators of PharmGKB and CPIC (https://www.pharmgkb.org/page/pgxGeneRef). We focused on the 11 clinically important pharmacogenes *CYP2C19*, *CYP2C9*, *CYP2D6*, *CYP3A5*, *CYP4F2*, *DPYD*, *IFNL3*, *SLCO1B1*, *TPMT*, *UGT1A1*, and *VKORC1*. CPIC gene-specific tables of allele definitions, functionality, phenotype, and frequency (downloaded on 17 September 2017) were used to first detect the pair of particular alleles for each gene and sample, and then estimate the corresponding phenotype. Of the 356 variants in the CPIC tables used for defining the star alleles of these genes, 356 (100%), 307 (86%), 101 (28%), and 31 (9%) could potentially be directly genotyped by the GS, ES, GSA, and OMNI platforms, correspondingly, if the data sets contained individuals carrying the variants. However, as the allele definition tables are large and not accompanied with decision trees for variant prioritization, direct uncurated application of the existing tables would result in a high proportion of ambiguous calls, mainly caused by haplotypes composed of variants that match several star alleles, or no matches in cases where the allele definition tables contained too many irrelevant variants. Therefore, we first pruned the allele definition tables manually based on scientific evidence for functional effects of the variants and removed duplicate as well as proxy alleles.

First, we removed star alleles with unknown function or with unnecessary proxies (mostly suballeles) from *CYP2C19* (*35), *CYP2D6* (68 alleles, mostly suballeles), *DPYD* (*9A and *9B combined into *9), and *SLCO1B1* (32 alleles with unknown function); see Table S1 for details on reasons for variant exclusion. For CYP2C19*2, which is defined by two variants that are in complete linkage disequilibrium (*r*^*2*^ = 1.0), we found that a single variant (*rs4244285*) is sufficient for its detection. Finally, we disregarded *CYP2D6* star alleles requiring gene deletions (*5) or duplications (star alleles with suffix “xN”) in the OMNI and ES data sets, because detection of copy numbers of *CYP* genes is limited on these platforms. These filtering steps resulted in 239 variants remaining in the allele definition tables. The final number of candidate star alleles that remained for each gene and data source after filtering is summarized in Table S2.

Because there is no specific allele definition table for typing of *HLA* alleles, we could not use the same pipeline for this region. However, to provide an overview of the relevant functional variability of the *HLA* region in the studied population, we used the SNP2HLA tool in the major histocompatibility complex region for the detection of *HLA* variants among individuals with GS data.

### Pipeline for star allele and phenotype detection and analysis

For all of the samples we detected their possible star alleles by checking each star allele given in the allele definition tables one by one and testing for the presence of defining variants for each allele. As this could result in several matching alleles due to missing data at certain positions, we found it reasonable to allow nonfunctional alleles to override other alleles. Therefore, we first checked for the presence of variants defining nonfunctional star alleles only, and if none of these matched, we tested the remaining star alleles. In ideal cases, only a single star allele matched (“single match”) (Fig. 1b). In some complex cases, the detected variants correspond to several star alleles (“ambiguous call”). Again, we reasoned that if one of the matching alleles was defined as “decreased function,” we could let this override “normal function” alleles. Cases where an individual carried a combination of variants that did not have any corresponding star allele in the reference table were defined as “no match.”

For detection of *CYP2D6* large deletions, large duplications, and multiallelic copy-number variants (CNVs) in GS data we used the Genome STRiP CNV discovery pipeline (version 2.00.1611) (ref. ^14^) for 2269 deeply sequenced genomes. For detection of CNVs in the array data we used the PennCNV software. We excluded individuals with <98% call rate, standard deviation of log R ratio >0.3, absolute waviness factor >0.05, and number of CNVs >100. We ended up with CNVs for a set of 30,100 individuals that had been genotyped on the GSA. We could not detect CNVs on the OMNI array because it only contains four markers covering the *CYP2D6* gene. We used estimated information of *CYP2D6* CNVs together with our developed pipeline for star allele detection to assign *CYP2D6* star allele diplotypes. For detected duplications, we assumed an allele of the order *2>*1>*4 to be duplicated, based on previous duplication frequencies in Europeans.^15^

For each sample, all possible diplotypes were constructed based on detected star alleles. The subsequent phenotype calling was based on PharmGKB’s diplotype-to-phenotype mapping tables.

The described pipeline was written as a custom Python script (available upon request). The calculation part of the haplotype and diplotype detection was run in the High Performance Computing Center at the University of Tartu. The results of the allele, effect, and phenotype detection were analyzed in R^16^ version 3.2.3 using the following packages: dplyr, reshape2 and ggplot2.

Finally, we compared the obtained phenotype predictions with previously reported allele and phenotype frequencies of Caucasians (Europeans + North Americans). For this comparison, each sample was used once; GS data was preferred over ES, GSA, and OMNI. As a result, 2420, 2356, 33,086, and 6586 samples were used from GS, ES, GSA, and OMNI data correspondingly. The results of 1661 samples that were sequenced/genotyped by more than a single method are compared in Note S2. For the GS data, we also validated the nonstructural star alleles and diplotypes of *CYP2D6* using an external tool (Astrolabe, previously called Constellation).^17^ Furthermore, we estimated the potential clinical impact of the variants based on drug consumption statistics in Estonia (Annual Statistical Reports of the State Agency of Medicines), Finland (The Social Insurance Institution of Finland), Sweden (The National Board of Health and Welfare of Sweden), Denmark (Statistics on the Total Sales of Medicines in Denmark), and Norway (Drug Consumption in Norway 2012–2016).

The study was conducted in accordance with good ethical standards, and was approved by the Ethics Committee of the University of Tartu (protocol number 234/T-12).

## RESULTS

### Comparison of allele calls across four different genotyping platforms

We compared the pharmacogenomic predictions for biobank participants genotyped with any of four different microarray or sequencing platforms (Fig. 1). Using the existing data sets combined with genotype imputation and phasing, we identified 100, 64, 61, and 43 different variants using GSA, OMNI, GS, and ES, respectively. Note that the larger number of variants in the microarray data is driven by more samples having been genotyped than sequenced. We assessed the imputation accuracy to be extremely high (99.96% matching genotype calls), which is described in further detail in Note S2.

**Fig. 2:**
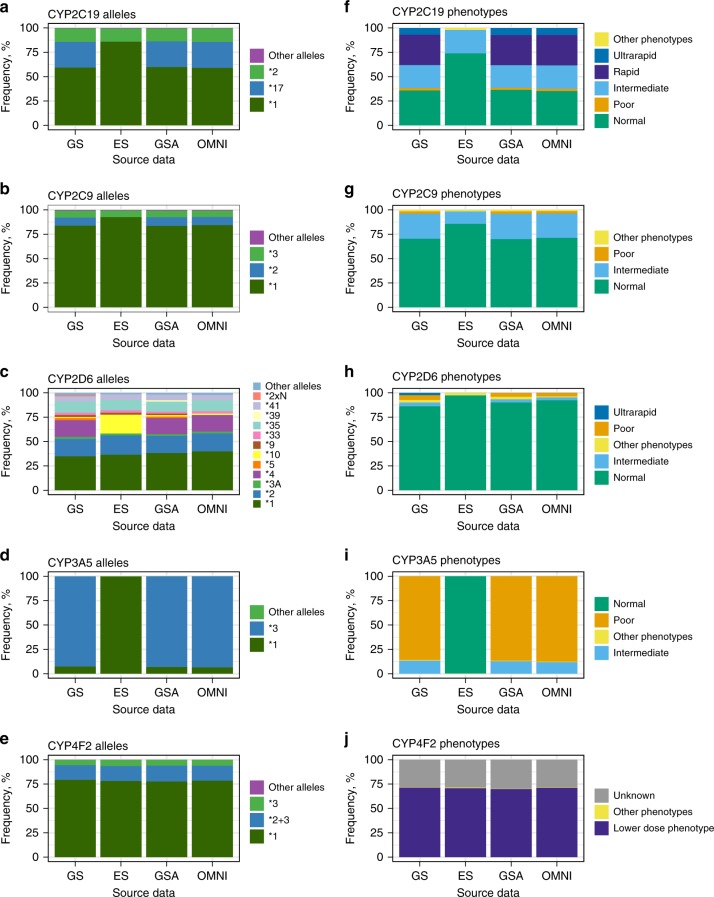
**Frequencies of predicted alleles and phenotypes by CYP gene and method.** The results for OMNI and GSA are based on imputed microarray genotype data. The decision to assign an allele a wild-type status (*1) is based upon a genotyping test that interrogates only the most common and already-proven sites of functional variation. In human DNA, it is always possible that a new, previously undiscovered (and therefore uninterrogated) site of variation may confer loss of function in an individual, and thus lead to the rare possibility of a nonfunctional allele being erroneously called as wild type. Alleles and phenotypes with frequencies below 2% are marked as “Other” for better visualization. *ES* exome sequencing, *GS* genome sequencing, *GSA* Global Screening Array, *OMNI* HumanOmniExpress.

**Fig. 3:**
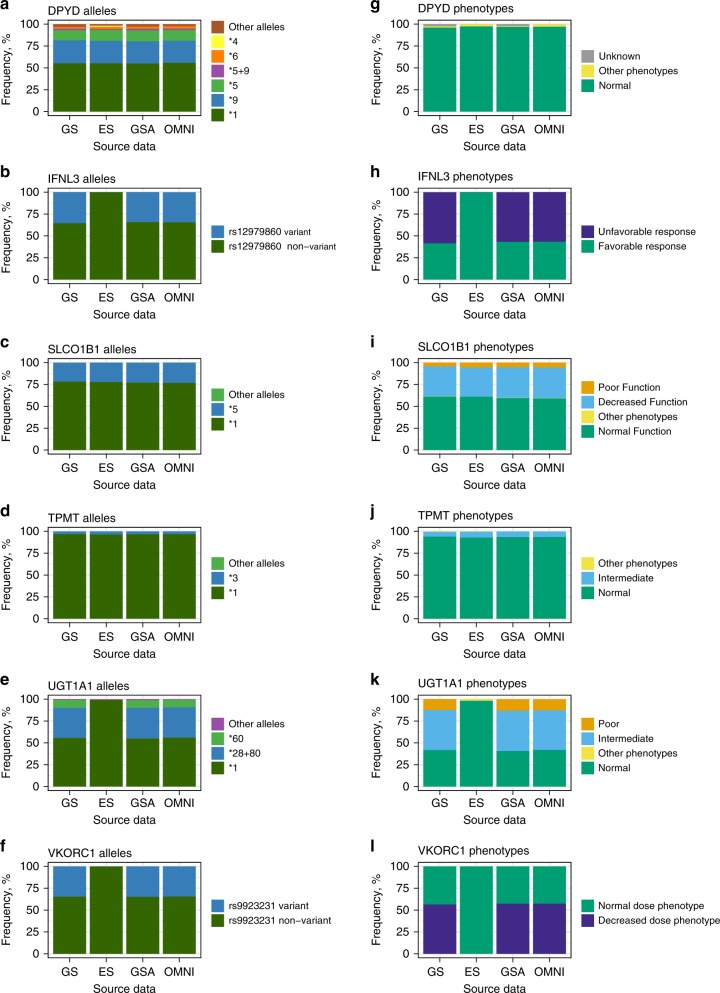
**Frequencies of predicted alleles and phenotypes by gene and method for non-CYP genes.** The results for OMNI and GSA are based on imputed microarray genotype data. The decision to assign an allele a wild-type status (*1) is based upon a genotyping test that interrogates only the most common and already-proven sites of functional variation. In human DNA, it is always possible that a new, previously undiscovered (and therefore uninterrogated) site of variation may confer loss of function in an individual, and thus lead to the rare possibility of a nonfunctional allele being erroneously called as wild type. Alleles and phenotypes with frequencies below 2% are marked as “Other” for better visualization. *ES* exome sequencing, *GS* genome sequencing, *GSA* Global Screening Array, *OMNI* HumanOmniExpress.

Overall, the proportion of calls with no matches is very low in all data sets, ranging from 0.01% to 0.05%. Ambiguous call frequencies ranged from 0.08% to 0.12%, mostly caused by difficulties of distinguishing between *5/*6/*9 in *DPYD*. However, as all three alleles have normal function, these ambiguous calls did not affect the phenotype predictions. For the remaining 99.8% of the samples, star alleles for each gene were unambiguously detected. The most notable novel finding is in *CYP4F2*, where in addition to *2 and *3 (both defined by single variants) both variants *2 *+* *3* are detected on the same allele in 15.5% of the samples. Figure 2a–e and Fig. 3a–f show the frequencies of the detected star alleles by genotyping method. The full table of the frequencies of the detected alleles, including ambiguous calls and no matches, is provided as Table S3.

The figures clearly illustrate that the microarray-based methods combined with imputation produce results that are very similar to GS. In contrast, ES does not allow the detection of 11 star alleles that are defined by variants outside the coding regions (see Table S2 for details). In addition, CYP2C9*2 and CYP2D6*4 could not be detected either, because the defining variants *rs1799853* and *rs3892097* did not pass quality control (QC).

To illustrate the proportion of rare variants detected in the 11 pharmacogenes under study, we assessed the frequencies of loss-of-function (LoF) and missense variants detected by GS and ES in these genes (Table 1, details in Table S4). Altogether 89% (*n* = 198) of the variants that we identified as putatively LoF or missense in the 11 pharmacogenes were rare with minor allele frequency (MAF) <1%, and 52% (*n* = 102) of the variants were novel.

**Table 1:** The frequencies of predicted functional variants in 12 pharmacogenes (including HLA) identified in sequencing data and frequencies of detected copy-number variants in *CYP2D6*

Variation in 11 pharmacogenes detected by sequencing		
	*n*	%
Loss-of-function and missense	198	n/a
Missense	188	94.95
Loss-of-function	10	5.05
Known variants	96	48.48
Novel variants	102	51.52
MAF >5%	21	10.61
1% ≤ MAF <5%	11	5.56
0.1% ≤ MAF <1%	34	17.17
MAF <0.1%	132	66.67
**HLA alleles of high-risk phenotypes** ^a^ **detected by genome sequencing data**	***n***	**%**
Individuals with data of typing HLA alleles	2243	100
Individuals with presence of at least one HLA-B*57:01 allele	105	4.68
Individuals with presence of at least one HLA-B*58:01 allele	32	1.43
Individuals with presence of at least one HLA-B*15:02 allele	0	0
Individuals with presence of at least one HLA-A*31:01 allele	109	4.86
***CYP2D6 *** **copy-number variants detected by genome sequencing and microarray data**	***n***	**%**
Number of individuals	32,369	n/a
Individuals with CYP2D6 deletion	1073	3.31
Individuals with CYP2D6 duplication	257	0.79

*MAF* minor allele frequency.

^a^Four high-risk phenotypes of the HLA region covered with Clinical Pharmacogenetics Implementation Consortium (CPIC) guidelines.

### Pharmacogenetic phenotype frequencies

Next, we used the called star alleles to derive actionable phenotypic predictions for all 11 analyzed genes (Fig. 2f–j, Fig. 3g–l). All diplotype frequencies are listed in Table S5 and phenotype frequencies in Table S6. As with the star allele calling the results are very similar for the different methods, with the exception of ES. From the perspective of implementing pharmacogenomics in the clinic, it is most crucial to accurately predict high-risk phenotypes, i.e., individuals with other than normal drug metabolizing phenotypes and who therefore require higher or lower dosing of a medication. Again, we observe in Fig. 2–3 and S1 that ES data is least suitable for pharmacogenomics because a high proportion of high-risk phenotypes remain undetected, except for *CYP4F2*, *DPYD*, *SLCO1B1*, and *TPMT*. For *CYP3A5*, ES does not interrogate the common (MAF >90% in Europeans) intronic splice variant CYP3A5*3 (rs776746) and thus incorrectly annotates all individuals with the high-risk *1/*1 diplotype. Therefore, we excluded ES results from the subsequent analyses where we evaluated the presence of high-risk phenotypes. In the 42,092 individuals under study, we found that nonstandard dosing information is required based on at least one gene for 99.8% of the individuals.

The SNP2HLA tool allowed us to call 6-digit *HLA* haplotypes in the GS data set. Of the four high-risk phenotypes of the *HLA* region covered with CPIC guidelines we detected HLA-B*57:01, HLA-B*58:01, and HLA-A*31:01 alleles with carrier frequencies of 4.7%, 1.4%, and 4.7%, respectively (Table 1). Because we were only able to call *HLA* alleles in the GS data, we could not compare the results between the different platforms.

We compared the results with frequencies reported in PharmGKB and by Muir et al.^18^ (see Table S6 for details). In general, the frequencies of the detected alleles and phenotypes correspond to what has been reported previously. However, slight differences appear. For instance, there are significantly more *CYP2C19* rapid and ultrarapid metabolizers among Estonians (30.8% and 7.3%, respectively) compared with other Europeans (26.9% and 4.6%, respectively, *p* values of one-proportion *z*-test 1.64 × 10^−72^ and 1.53 × 10^−155^). Because *CYP2D6* is the only gene with CNVs included in the allele definition tables in PharmGKB, we detected *CYP2D6* CNVs in the GS and GSA data (Table 1, details in Table S4). In addition, we compared the results we obtained for *CYP2D6* using our approach with those obtained using a published tool (Astrolabe). In 98% of the samples the detected alleles were identical; the discrepancies were mostly caused by CYP2D6*59, which is included in Astrolabe. We excluded this star allele from our candidate list due to sparse information about its suggested decreased function.^15^ The overview of the comparison is illustrated in Figure S2.

### Relevance of detected phenotypes

Based on the dosing guidelines of CPIC, genetic variations in the 11 genes under study are associated with response to at least 32 currently prescribed medications (Table S7). *CYP2C19* affects the metabolism of drugs frequently used in the clinic,^19^ and CPIC dosing guidelines are currently available for ten active substances of these drugs. For this gene, we found that 2.2% of individuals in the studied cohort were poor metabolizers and 30.8% and 7.3% rapid or ultrarapid metabolizers, respectively (Table 2). Thus, in total, 40.4% of the individuals in the Estonian population may be at risk for unwanted outcomes or may need dosing adjustments when prescribed any of these ten drugs. As shown in Table 2, the combined intake of medications associated with *CYP2C19* ranges from 17.62 to 66.83 DDD/1000 inhabitants per day in the Nordic countries and Estonia (data from Annual Statistical Reports, 2016).

**Table 2:** Frequencies of predicted high-risk phenotypes within the studied cohort (GS, GSA, and OMNI data combined) and gene-related drug consumption statistics in European Nordic countries

Gene	Phenotype	% of individuals (phenotype, source)			% of individuals (gene total)	Number of drug active substances affected	DDD^a^/1000 inhabitants, (min–max)^b^
		GS	GSA	OMNI			
*CYP2C19*	Intermediate metabolizer	23.6	23.2	24.0	63.7	10	17.62–66.83
	Poor metabolizer	2.44	2.16	2.34			
	Rapid metabolizer	31.2	30.7	31.2			
	Ultrarapid metabolizer	6.86	7.40	7.23			
*CYP2C9*	Intermediate metabolizer	25.8	26.1	25.1	28.4	2	7.08–16.26
	Poor metabolizer	2.40	2.49	2.32			
*CYP2D6*	Intermediate metabolizer	3.93	3.26	2.96	7.65	16	9.16–15.92
	Poor metabolizer	4.96	4.07	3.67			
	Ultrarapid metabolizer	2.36	0.27	0			
*CYP3A5*	Intermediate metabolizer	13.5	12.8	11.9	13.2	1	0–0.5
	Normal metabolizer	0.62	0.51	0.55			
*CYP4F2*	Higher dose phenotype	0.29	0.36	0.33	70.5	1	7.02–16.04
	Increased CYP4F2 activity	0.04	0.02	0.03			
	Lower dose phenotype	71.3	69.8	71.3			
*DPYD*	Intermediate metabolizer	1.36	0.90	0.87	0.92	3	0
	Poor metabolizer	0	0.006	0			
*IFNL3*	Unfavorable response	58.5	56.7	56.7	56.8	3	0–0.23
*SLCO1B1*	Decreased function	34.0	34.9	35.2	40.1	1	6.13–62.9
	Poor function	4.38	5.24	5.47			
*TPMT*	Intermediate metabolizer	5.54	6.37	6.33	6.40	3	0.32–1.41
	Poor metabolizer	0.21	0.07	0.08			
*UGT1A1*	Intermediate metabolizer	45.9	46.2	45.3	59.0	2	0–0.09
	Poor metabolizer	12.3	13.1	12.6			
*VKORC1*	Decreased dose phenotype	56.5	57.5	57.5	57.4	1	7.02–16.04

*GS* genome sequencing, *GSA* Global Sequencing Array, *OMNI* HumanOmniExpress.

^a^Drug daily dosage.

^b^Min–max among Estonia, Finland, Sweden, Denmark, Norway.

Further, we also investigated the number of individuals with high-risk variants who had been prescribed drugs associated with the specific genes. As seen in Table S7, as many as 12,254 individuals in the Estonian Biobank have actually had a prescription of at least one drug linked to *CYP2C19*. Of these, 9977 were analyzed in our study (GS, GSA, and OMNI) and 40.7% of them (*n* = 4059) are *CYP2C19* poor, rapid, or ultrarapid metabolizers, and therefore may have needed dosing adjustments to improve treatment outcome. Based on the Annual Statistics of the Estonian Agency of Medicines, on average almost 5.5% (55 DDD/1000 inhabitants/day) of individuals in the population use at least 1 of the 32 drugs associated with the studied genes on a daily basis. For several Nordic countries, the numbers are even higher; the highest being for Denmark with on average 15.8% of individuals in the population (158.2 DDD/1000 inhabitants/day) (Table 2, Table S7). Thus, existing data of biobank participants can be an untapped resource for improved and more cost-effective recommendations for drug treatment by translating existing genotype/phenotype data of pharmacogenes into guiding prescription recommendations. This illustrates the enormous innovative potential of biobanks in the whole process of the implementation of pharmacogenomics.

## DISCUSSION

In this study, we assessed the systematic detection of pharmacogenetic star alleles for Biobank participants genotyped on different microarray or sequencing platforms. As most of the pharmacogenes have star alleles defined by several variants that all need to be on the same parental allele, a crucial step in the process was genotype phasing prior to analysis. Although the PharmGKB tables for defining star alleles have been thoroughly curated, prefiltering of the allele definition tables, as described in the Methods section, was essential for efficient detection of star alleles. Many of the allele definitions include additional variants beyond the variant(s) causing the functional effects, which can compromise allele calling when searching for perfect matches. For example, in the original *SLCO1B1* star allele definition table, 20 of 37 alleles require the occurrence of several variants on the same allele, but in our data set of 44,448 individuals, only a subset of these were actually detected on the same alleles, ruling out all possible star alleles and subsequently leading to “no matches” without prior filtering. The same applies for *CYP2D6*, where less than half of the alleles are currently of relevance^20^ and including too many unvalidated alleles would only result in unknown phenotypes. Challenges with these definition tables have been observed by others as well with an additional remark that the tables do not contain all of the alleles that are common in respective populations.^15,21^

We found that 89% of the variants called in the genome and exome sequencing data that are predicted to have functionally deleterious effects are rare, with MAF <1%. The proportion of rare variants detected in pharmacogenes has increased with the growing numbers of NGS studies.^3,22–28^ Including rare variants with unknown function in pharmacogenetic reporting is objectionable because their function and relevance are generally not well validated^2,9^ and care must be taken when including these in clinical implementation. However, including rare variants in test panels and collecting data on these variants is still valuable for further research and development projects. In the absence of experimental characterization data, the functional impact of variants can be predicted using computational methods, which are getting more and more precise with the increase in data that can be used for validation.^29,30^

Our comparison of different genotyping and sequencing platforms marks GS as the gold standard and the most comprehensive technology for detection of both rare and common functional alleles. It also highlights a known major shortcoming of ES for pharmacogenetic applications. Important alleles defined by variants in introns or promoters, such as CYP2C19*17 or CYP3A5*3, are not interrogated by ES and thus lead to drastically different pharmacogenetic recommendations that affect 13 medications according to CPIC guidelines. Unlike microarray data, ES data cannot be subjected to classical imputation due to large gaps in the data. These problems could be overcome by combining ES with customized capture probes, or simply replacing ES with custom panels such as PGRNseq,^25^ to provide a comprehensive cost-effective implementation of pharmacogenomics compared with GS.^31^ However, when the focus is exclusively on predefined alleles, genotyping arrays, which are currently at least ten times cheaper than ES or GS, are clearly a more cost-effective alternative that can generate results surprisingly similar to those of GS. The OMNI array used in our study unfortunately does not allow the detection of *CYP2D6* copy number, which is the greatest but still limited drawback when compared with GS and the GSA (Table 2). As cost-effectiveness is still considered a major barrier for the clinical implementation of pharmacogenetics,^32^ our data suggests that current genotyping microarrays might constitute the most cost-effective technology with acceptable accuracy. Several studies have found preemptive pharmacogenetic testing cost efficient, with per-patient savings ranging from USD5962 to USD10,667 (refs. ^33–35^), despite the reported costs of pharmacogenetic testing to be over USD2000 (ref. ^33^). Thus, both genotyping per se and developing tools for the translation of preexisting genome-wide genotype data into clinical recommendations can be considered very reasonable health-care investments.

In conclusion, as the number of sequenced and genotyped participants in biobanks and clinical settings is growing rapidly in several countries, we now have a large amount of genetic information that could be translated into clinically actionable decisions tailoring medical therapy in the near future. By leveraging the existing genotype data of 44,448 individuals in the Estonian Biobank, we were able to determine that microarrays with imputed variants are a highly cost-effective tool for identifying thousands of individuals who need dosing adjustments for commonly prescribed drugs. In total, we found that as many as 99.8% of the individuals have a high-risk phenotype requiring a nonstandard dosing of a medication based on at least one gene, which is even larger than shown before. Our approach of trying to define all possible star alleles in the majority of genes with CPIC guidelines allowed us to reveal the many challenges that arise in this process. The most crucial next steps we suggest are further revision of star allele definition tables based on existing haplotypes in different populations, an additional level of decision trees to prioritize variants causing nonfunctional alleles, and restricting the inclusion of rare alleles to functionally validated variants. We are confident that such developments built into automated decision support for clinicians will allow the implementation of pharmacogenomics at the point of care in a multidisciplinary manner^36^ and with greater impact.

## Electronic supplementary material

Supplementary FigS1

Supplementary FigS2

Supplementary Note S1

Supplementary Note S2

Supplementary Table S1

Supplementary Table S2

Supplementary Table S3

Supplementary Table S4

Supplementary Table S5

Supplementary Table S6

Supplementary Table S7

Supplementary Legends
