# Supplementary material for: Translating genotype data of 44,000 biobank participants into clinical pharmacogenetic recommendations: challenges and solutions
Source: Genet Med. 2018 Oct 16;21(6):1345–54. doi: 10.1038/s41436-018-0337-5 (PMC6752278; doi:10.1038/s41436-018-0337-5)
Supplement: Supplementary file 1 — Supplementary FigS1 [file 41436_2018_337_MOESM1_ESM.docx]

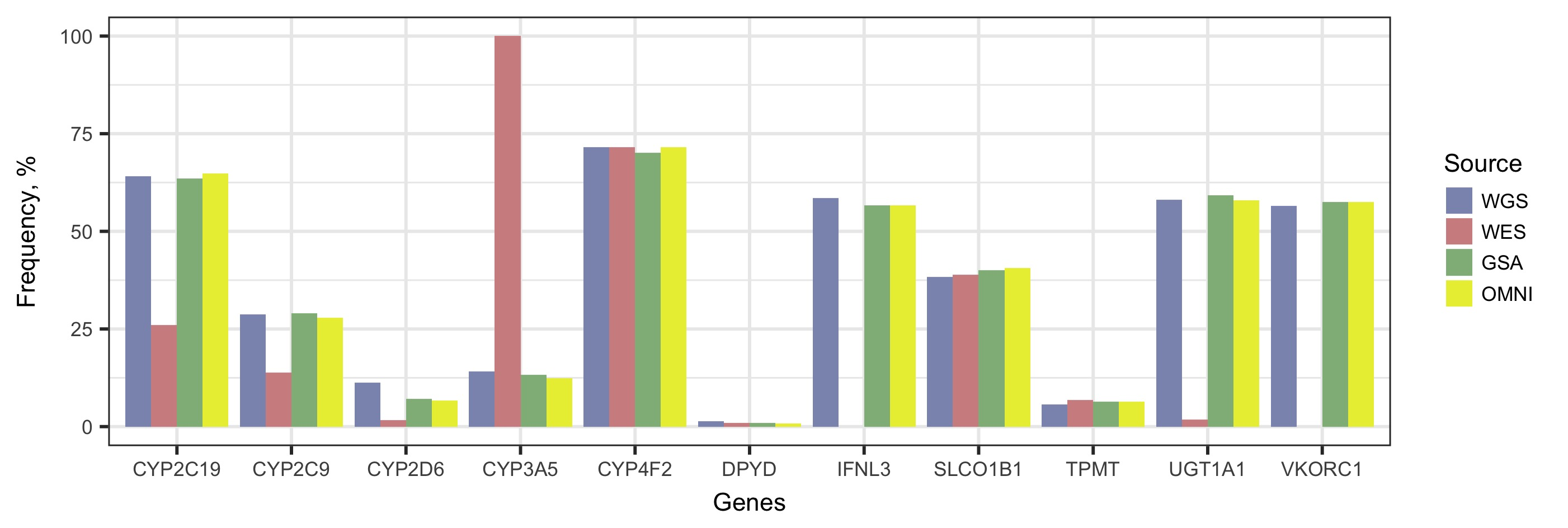


**Figure S1. Fraction of high risk phenotypic predictions by gene and method.** High risk phenotypes are defined as those that differ from normal and unknown phenotypes and would require a different drug dosing or recommendation.
