# Supplementary material for: Translating genotype data of 44,000 biobank participants into clinical pharmacogenetic recommendations: challenges and solutions
Source: Genet Med. 2018 Oct 16;21(6):1345–54. doi: 10.1038/s41436-018-0337-5 (PMC6752278; doi:10.1038/s41436-018-0337-5)
Supplement: Supplementary file 2 — Supplementary FigS2 [file 41436_2018_337_MOESM2_ESM.docx]

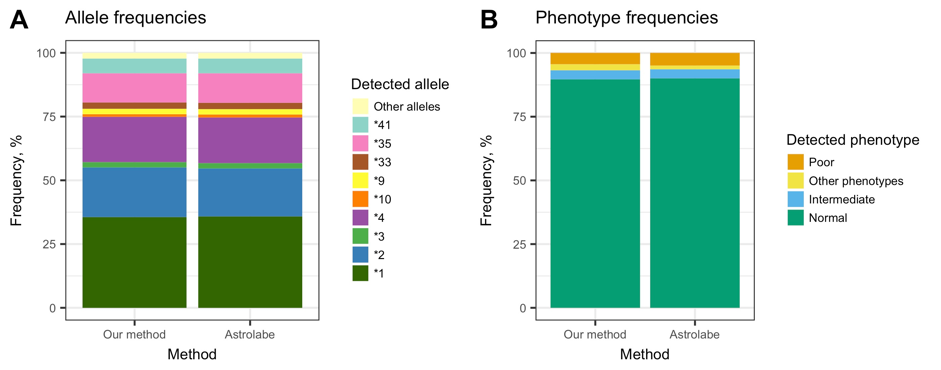


**Figure S2.** *CYP2D6* allele and phenotype frequencies in WGS derived by two methods (our method, Astrolabe)
