# Supplementary material for: Translating genotype data of 44,000 biobank participants into clinical pharmacogenetic recommendations: challenges and solutions
Source: Genet Med. 2018 Oct 16;21(6):1345–54. doi: 10.1038/s41436-018-0337-5 (PMC6752278; doi:10.1038/s41436-018-0337-5)
Supplement: Supplementary file 3 — Supplementary Note S1 [file 41436_2018_337_MOESM3_ESM.docx]

## Supplementary Note S1. Preparation of genetic data

Sequenced reads were aligned against the GRCh37/hg19 version of the human genome reference using BWA-MEM^1^ v0.7.7; PCR duplicates were marked using Picard (<http://broadinstitute.github.io/picard>) v1.136, and the Genome Analysis Toolkit (GATK)^2^ v3.4-46 applied for further processing of BAM files and genotype calling. All insertion-deletions (indels) in the Variant Call Format (VCF)^3^ were normalized and multiallelic sites split using bcftools (<https://samtools.github.io/bcftools/bcftools.html>).

The following genotypes were set to missing: genotype quality <20, read depth >200 for WGS and <8 for WES, allele balance <0.2 or >0.8 for heterozygous calls. The GATK’s Variant Quality Score Recalibration (VQSR) metric was used to filter variants with a truth sensitivity of 99.8% for SNVs and of 99.9% for indels. Furthermore, variants with inbreeding coefficient <-0.3, quality by depth <2 for SNVs and <3 for indels, call rate < 95%, or Hardy-Weinberg equilibrium (HWE) P-value <1×10^-6^ were excluded.

A population-specific imputation reference panel^4^ contains the same subset of WGS individuals, but slightly different quality control parameters were used: only unrelated individuals were considered (IBD proportion <0.1); we excluded variants with call rate <90%, HWE P-value <1×10^-9^, multi-allelic variants, and low-complexity regions^5^. Finally, WGS data of 2279 Estonians and 1856 Finns were merged, where quality control was performed independently for each study.

The genotype calling for the GSA and OMNI arrays was performed using Illumina’s GenomeStudio V2010.3 software. The genotype calls for rare variants on the GSA array were corrected using the zCall software (version May 8th, 2012). After variant calling, the data was filtered using PLINK (v.1.90)^6^ by sample (call rate >95%, no sex mismatches between phenotype and genotype data, heterozygosity < mean +-3 SE) and marker-wise (HWE p-value >1×10^-6^, call rate >95%, and for the GSA array additionally by Illumina GenomeStudio GenTrain score >0.6, Cluster Separation Score >0.4). Before the imputation, variants with MAF <1% and C/G or T/A polymorphisms as well as indels were removed, as these genotype calls do not allow precise phasing and imputation.

References

1. Li, H. & Durbin, R. Fast and accurate short read alignment with Burrows-Wheeler transform. *Bioinformatics* **25,** 1754–1760 (2009).

2. McKenna, A. *et al.* The Genome Analysis Toolkit: a MapReduce framework for analyzing next-generation DNA sequencing data. *Genome Res.* **20,** 1297–303 (2010).

3. Danecek, P. *et al.* The variant call format and VCFtools. *Bioinformatics* **27,** 2156–8 (2011).

4. Mitt, M. *et al.* Improved imputation accuracy of rare and low-frequency variants using population-specific high-coverage WGS-based imputation reference panel. *Eur. J. Hum. Genet.* **25,** 869–876 (2017).

5. Li, H. & Wren, J. Toward better understanding of artifacts in variant calling from high-coverage samples. *Bioinformatics* **30,** 2843–2851 (2014).

6. Purcell, S. *et al.* PLINK: A Tool Set for Whole-Genome Association and Population-Based Linkage Analyses. *Am. J. Hum. Genet.* **81,** 559–575 (2007).
