# Supplementary material for: Translating genotype data of 44,000 biobank participants into clinical pharmacogenetic recommendations: challenges and solutions
Source: Genet Med. 2018 Oct 16;21(6):1345–54. doi: 10.1038/s41436-018-0337-5 (PMC6752278; doi:10.1038/s41436-018-0337-5)
Supplement: Supplementary file 4 — Supplementary Note S2 [file 41436_2018_337_MOESM4_ESM.docx]

## Supplementary Note S2. Variant calls were highly accurate

For 49 variants, the genotypes were derived both by direct detection on a microarray and by imputation. For assessing the imputation quality, we first compared these two genotypes to each other. In total, only 631 genotypes out of 1.68 million (0.038%) had different calls. All the differences occurred in 30 out of the 49 investigated variants, and in all these cases we preferred the original genotype call instead of the imputed one.

Second, 1,661 samples in our data that had been sequenced/genotyped by more than a single method, allowed us to compare the derived results between these platforms and detect possible discrepancies. Most (n=1,296, 78%) of the individuals had both WGS and OMNI data available. For those, we conducted a variant-by-variant comparison of WGS and imputed OMNI data, checking 58 SNPs in total. Out of 75 thousand tests 99.8% imputed genotypes matched to WGS genotypes and only 115 discrepancies were detected (0.15% of all, 1.6% out of non-reference genotypes). Below is a full table of the results.

|  | **Number of matches** | **Number of non-REF matches** | **Number of mismatches** | **Mismatches %** |
| --- | --- | --- | --- | --- |
| rs1080985 | 1291 | 116 | 5 | 0.387 |
| rs35742686 | 1295 | 0 | 1 | 0.077 |
| rs4124874 | 1294 | 250 | 2 | 0.155 |
| rs28624811 | 1293 | 174 | 3 | 0.232 |
| rs2291075 | 1292 | 201 | 4 | 0.31 |
| rs1080995 | 1293 | 173 | 3 | 0.232 |
| rs9332239 | 1296 | 1 | 0 | 0 |
| rs1080983 | 1293 | 170 | 3 | 0.232 |
| rs11045819 | 1296 | 12 | 0 | 0 |
| rs28371705 | 1293 | 30 | 3 | 0.232 |
| rs1081003 | 1295 | 2 | 1 | 0.077 |
| rs769258 | 1291 | 15 | 5 | 0.387 |
| rs12979860 | 1294 | 177 | 2 | 0.155 |
| rs1801159 | 1294 | 18 | 2 | 0.155 |
| rs9923231 | 1294 | 166 | 2 | 0.155 |
| rs79292917 | 1295 | 0 | 1 | 0.077 |
| rs1142345 | 1296 | 2 | 0 | 0 |
| rs1058164 | 1292 | 393 | 4 | 0.31 |
| rs28371717 | 1295 | 2 | 1 | 0.077 |
| rs1801158 | 1294 | 0 | 2 | 0.155 |
| rs74644586 | 1293 | 173 | 3 | 0.232 |
| rs28371699 | 1293 | 396 | 3 | 0.232 |
| rs3892097 | 1295 | 32 | 1 | 0.077 |
| rs12248560 | 1294 | 81 | 2 | 0.155 |
| rs28371703 | 1295 | 31 | 1 | 0.077 |
| rs1057910 | 1295 | 4 | 1 | 0.077 |
| rs76312385 | 1293 | 174 | 3 | 0.232 |
| rs1080996 | 1293 | 172 | 3 | 0.232 |
| rs1080989 | 1295 | 39 | 1 | 0.077 |
| rs4149057 | 1293 | 374 | 3 | 0.232 |
| rs2108622 | 1292 | 49 | 4 | 0.31 |
| rs2267447 | 1295 | 36 | 1 | 0.077 |
| rs887829 | 1295 | 157 | 1 | 0.077 |
| rs12169962 | 1293 | 172 | 3 | 0.232 |
| rs121909011 | 1295 | 0 | 1 | 0.077 |
| rs150163869 | 1296 | 1 | 0 | 0 |
| rs12769205 | 1294 | 31 | 2 | 0.155 |
| rs28371704 | 1295 | 31 | 1 | 0.077 |
| rs16947 | 1293 | 174 | 3 | 0.232 |
| rs28588594 | 1293 | 39 | 3 | 0.232 |
| rs4149056 | 1294 | 60 | 2 | 0.155 |
| rs2306283 | 1292 | 197 | 4 | 0.31 |
| rs28371729 | 1295 | 0 | 1 | 0.077 |
| rs2842934 | 1291 | 752 | 5 | 0.387 |
| rs1065852 | 1295 | 39 | 1 | 0.077 |
| rs3918290 | 1295 | 0 | 1 | 0.077 |
| rs3093105 | 1293 | 19 | 3 | 0.232 |
| rs182132442 | 1295 | 0 | 1 | 0.077 |
| rs28371725 | 1296 | 9 | 0 | 0 |
| rs11045818 | 1296 | 12 | 0 | 0 |
| rs776746 | 1294 | 1107 | 2 | 0.155 |
| rs1800460 | 1296 | 1 | 0 | 0 |
| rs28371730 | 1293 | 166 | 3 | 0.232 |
| rs1799853 | 1295 | 8 | 1 | 0.077 |
| rs4244285 | 1294 | 34 | 2 | 0.155 |
| rs4149015 | 1295 | 13 | 1 | 0.077 |
| rs1801265 | 1296 | 115 | 0 | 0 |
| rs1135840 | 1292 | 396 | 4 | 0.31 |

Further, to assess the differences between the phenotype predictions of different platforms, we compared predicted phenotypes of these 1,661 samples also. We defined a discrepancy as the differences in diplotype or phenotype calling of the same sample and gene on different platforms (e.g *CYP2C19*17/*17* in one dataset and *CYP2C9*17/*2* in the other). As seen in the main text, WES performed relatively poorly, and therefore we left WES out of the result comparison (352 samples). We compared the results of 1,309 samples and only 71 samples (5.4%) had some discrepancies in the phenotype prediction results, predominantly in *CYP2D6*. This underlines the high quality and accuracy of pharmacogenetic variants obtained in research settings, as previously shown by others (PMID 28502727). The slight differences in OMNI and WGS results are expected, as OMNI predictions are made on partial genetic information only (measured+imputed) and in all other positions wild type is assumed. In addition, some imputation and phasing errors are possible.
