# Supplementary material for: Translating genotype data of 44,000 biobank participants into clinical pharmacogenetic recommendations: challenges and solutions
Source: Genet Med. 2018 Oct 16;21(6):1345–54. doi: 10.1038/s41436-018-0337-5 (PMC6752278; doi:10.1038/s41436-018-0337-5)
Supplement: Supplementary file 12 — Supplementary Legends [file 41436_2018_337_MOESM12_ESM.docx]

# Supplementary Information

**Note S1. Preparation of genetic data**

**Table S1. Star-alleleles that where filtered out in the pipeline**

**Table S2. Size of star allele definition tables**

**Note S2. Variant calls were highly accurate**

**Table S3. Frequencies of the detected alleles**

**Table S4. The frequencies of predicted functional variants in 12 pharmacogenes**

**Table S5. Frequencies of the detected diplotypes**

**Table S6. Frequencies of the detected phenotypes**

**Figure S1. Fraction of high risk phenotypic predictions by gene and method.** High risk phenotypes are defined as those that differ from normal and unknown phenotypes and would require a different drug dosing or recommendation.

**Figure S2. *CYP2D6* allele and phenotype frequencies in WGS derived by two methods (our method, Astrolabe)**

**Table S7. Drug usage in Northern European countries**
